# Affect-Focused Psychodynamic Internet-Based Therapy for Adolescent Depression: Randomized Controlled Trial

**DOI:** 10.2196/18047

**Published:** 2020-03-30

**Authors:** Karin Lindqvist, Jakob Mechler, Per Carlbring, Peter Lilliengren, Fredrik Falkenström, Gerhard Andersson, Robert Johansson, Julian Edbrooke-Childs, Hanne-Sofie J Dahl, Katja Lindert Bergsten, Nick Midgley, Rolf Sandell, Agneta Thorén, Naira Topooco, Randi Ulberg, Björn Philips

**Affiliations:** 1 Department of Psychology Stockholm University Stockholm Sweden; 2 Ersta Sköndal Bräcke University College Stockholm Sweden; 3 Department of Behavioural Sciences and Learning Linköping University Linköping Sweden; 4 Department of Clinical Neuroscience Karolinska Institute Stockholm Sweden; 5 Evidence Based Practice Unit Anna Freud National Centre for Children and Families London United Kingdom; 6 Department of Clinical, Educational and Health Psychology University College London London United Kingdom; 7 Vestfold Hospital Trust Oslo Norway; 8 Division of Mental Health and Addiction University of Oslo Oslo Norway; 9 Department of Psychology Uppsala University Uppsala Sweden; 10 Child Attachment and Psychological Therapies Research Unit Anna Freud National Centre for Children and Families London United Kingdom; 11 Department of Psychology Lund University Lund Sweden; 12 The Erica Foundation Stockholm Sweden; 13 Center for m2Health Palo Alto, CA United States; 14 Department of Psychiatry Diakonhjemmet Hospital Oslo Norway

**Keywords:** depressive disorder, adolescents, psychodynamic, internet-based treatment, treatment outcome, mobile phone

## Abstract

**Background:**

Adolescent depression is one of the largest health issues in the world and there is a pressing need for effective and accessible treatments.

**Objective:**

This trial examines whether affect-focused internet-based psychodynamic therapy (IPDT) with therapist support is more effective than an internet-based supportive control condition on reducing depression in adolescents.

**Methods:**

The trial included 76 adolescents (61/76, 80% female; mean age 16.6 years), self-referred via an open access website and fulfilling criteria for major depressive disorder. Adolescents were randomized to 8 weeks of IPDT (38/76, 50%) or supportive control (38/76, 50%). The primary outcome was self-reported depressive symptoms, measured with the Quick Inventory of Depressive Symptomatology for Adolescents (QIDS-A17-SR). Secondary outcomes were anxiety severity, emotion regulation, self-compassion, and an additional depression measure. Assessments were made at baseline, postassessment, and at 6 months follow-up, in addition to weekly assessments of the primary outcome measure as well as emotion regulation during treatment.

**Results:**

IPDT was significantly more effective than the control condition in reducing depression (*d*=0.82, *P*=.01), the result of which was corroborated by the second depression measure (*d*=0.80, *P*<.001). IPDT was also significantly more effective in reducing anxiety (*d*=0.78, *P*<.001) and increasing emotion regulation (*d*=0.97, *P*<.001) and self-compassion (*d*=0.65, *P*=.003). Significantly more patients in the IPDT group compared to the control group met criteria for response (56% vs 21%, respectively) and remission (35% vs 8%, respectively). Results on depression and anxiety symptoms were stable at 6 months follow-up. On average, participants completed 5.8 (SD 2.4) of the 8 modules.

**Conclusions:**

IPDT may be an effective intervention to reduce adolescent depression. Further research is needed, including comparisons with other treatments.

**Trial Registration:**

International Standard Randomised Controlled Trial Number (ISRCTN) 16206254; http://www.isrctn.com/ISRCTN16206254

## Introduction

According to the World Health Organization [1], adolescent depression is the fourth-leading cause of illness and disability among young people 15-19 years of age. Adolescent depression is associated with higher risk of adversity throughout life, including recurrent depressive episodes, other mental health issues, relational problems, and lower educational attainment [2]. Still, only a minority of young people suffering from depression seek and/or receive professional help [3,4]. Barriers to seeking help include practical issues of accessing services, perceived stigma and feelings of shame, desire to be self-reliant, and difficulties recognizing symptoms of mental health disorders [5].

Internet-based interventions may address some of the barriers to seeking and receiving treatment. For example, people living in rural areas may access care in their home rather than travelling potentially long distances to services [6]. Internet-based interventions may potentially also reach patients who otherwise would avoid seeking treatment due to social stigma [7]. Moreover, it has been suggested that internet-based treatments might engage adolescents with more severe symptoms who are reluctant to seek care [8]. Thus, making treatment more accessible for adolescents via the internet may lead to future health benefits in the population as it enables people to receive treatment at an earlier stage of their psychiatric illness.

To date, the literature on internet-based treatments for adolescent depression is lagging behind the research on adult populations [9]. Given the affinity young people have with communication via the internet, it is rather surprising that research on this group is scarce. The research that exists suggests that internet-based cognitive behavioral therapy (ICBT) is an effective treatment for adolescent and childhood psychopathology [10]. However, few studies have specifically targeted adolescent and/or childhood depression, and the ones that exist have shown a mixed pattern of findings [11-13]. Two recent randomized controlled trials (RCTs) indicated positive results for ICBT with enhanced synchronous chat support in the treatment of adolescent depression. ICBT led to a 50% or higher reduction of depressive symptoms in 42% of cases [14] and 46% achieved clinically significant improvement [15]. Although these results are highly promising, it is possible that some patients might be better helped by another type of treatment, meaning that treatment alternatives should be developed and tested.

One such treatment alternative is psychodynamic psychotherapy (PDT). Albeit not as extensively researched as cognitive behavioral therapy (CBT), several meta-analyses support the efficacy of PDT as a treatment for adults suffering from psychopathology [16,17]. The available research also supports PDT as an effective treatment for children and adolescents, but the research is much less robust due to the limited number of controlled studies [18,19]. Results from the largest RCT on psychotherapy for adolescents with depression suggested that PDT can be as clinically effective and cost-effective as CBT [20]. Recently, internet-based interventions based on psychodynamic principles, such as internet-based psychodynamic therapy (IPDT), have been developed and tested with promising results in adult populations, both as a transdiagnostic approach (eg, Johansson et al [21] and Zwerenz et al [22]) and an approach targeting specific mood and anxiety disorders [23-25]. However, to the authors’ knowledge, no study has yet tested the effects of an IPDT adaptation in an adolescent population suffering from depression.

Thus, this RCT aimed to evaluate a newly developed, affect-focused IPDT program for adolescent depression. The treatment was given as a guided self-help program with therapist support and weekly chat sessions and was compared to a control condition in which participants were given online therapist support. It was hypothesized that the treatment would be significantly more effective than the control condition for depression as well as for secondary outcomes, such as anxiety, emotion regulation, and self-compassion. Furthermore, treatment effects on depression and anxiety were investigated at a 6-month follow-up after treatment completion. As this was a trial of a newly developed treatment, acceptability and attitudes toward the treatment were also investigated.

## Methods

### Overview

This study was carried out in accordance with the Consolidated Standards of Reporting Trials (CONSORT) statement for clinical trials [26]. The ISRCTN (International Standard Randomised Controlled Trial Number) registration ID is 16206254. The trial was approved by the Regional Ethics Board of Stockholm, Sweden (number: 2018/2268-31/5). Participants received the treatment at no cost. Written informed consent was obtained from all participants via the online treatment platform. During the diagnostic interview, participants were also given the same information and had the opportunity to ask questions. Participants were not paid in any way for their participation or completion of measures. The project was undertaken by Stockholm University in collaboration with Linköping University.

### Recruitment and Participants

Adolescents were recruited via social media as well as through information via schools, youth centers, youth mental health care providers, and other similar locations during January and February 2019. Eligibility criteria were being aged 15-18 years; fulfilling a diagnosis of unipolar major depressive disorder according to DSM-5 (Diagnostic and Statistical Manual of Mental Disorders, Fifth Edition) criteria, as established by scoring at least 10 points on the Quick Inventory of Depressive Symptomatology for Adolescents (QIDS-A17-SR) [27]; and fulfilling criteria according to the Mini International Neuropsychiatric Interview 7.0 (MINI 7.0) [28]. Furthermore, participants had to have access to a computer, smartphone, or tablet with internet connection and had to be able to read, write, and speak Swedish without the aid of an interpreter. Exclusion criteria included a substantial risk of suicidality, as indicated by clearly stated intent and plans and/or earlier suicide attempts; partaking in other concurrent psychological treatment; psychotropic medication dosage not stable for at least 3 months; other primary diagnoses in need of other treatment; and current fulfilment of any of the following diagnoses: any psychotic disorder, bipolar I/II disorder, antisocial personality disorder, autism-spectrum disorder, or any substance use disorder.

### Procedure

**Figure 1:**
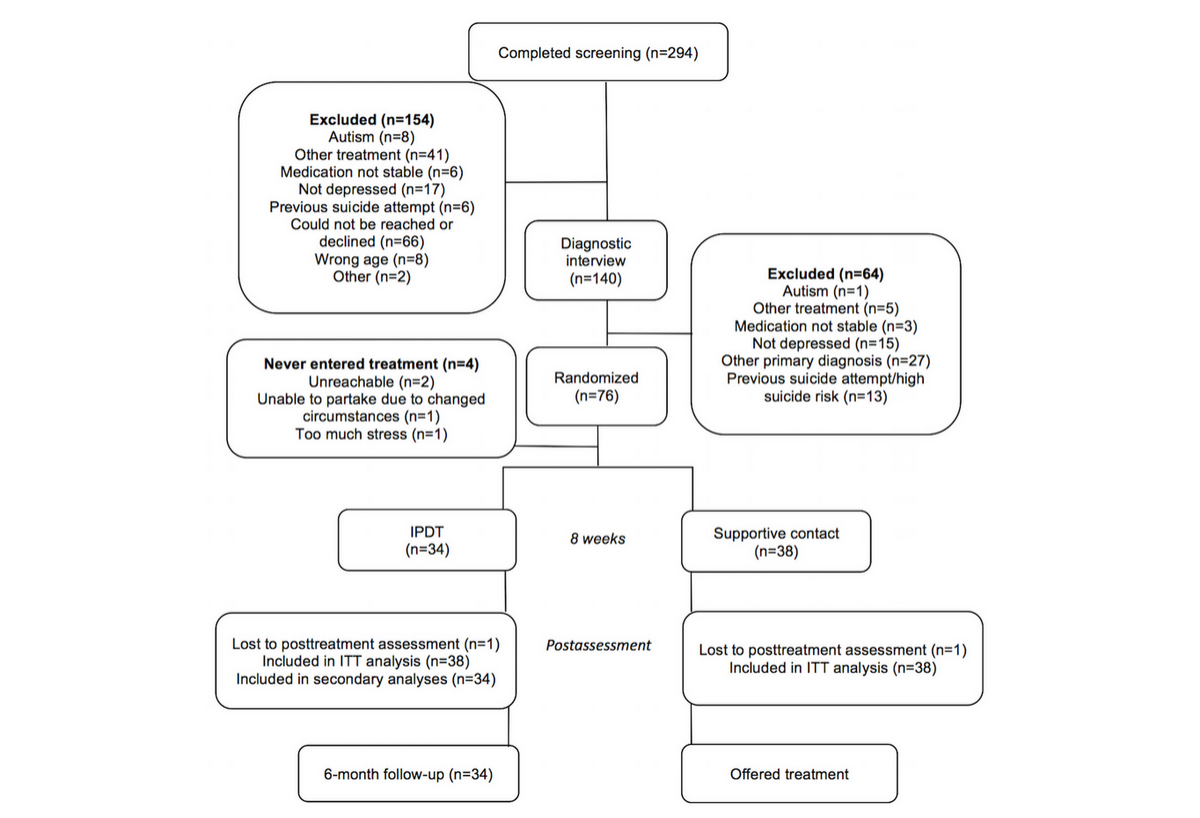
CONSORT (Consolidated Standards of Reporting Trials) flowchart. IPDT: internet-based psychodynamic therapy; ITT: intention-to-treat.

Potential participants were directed to a study website with information about the project and online registration. Informed consent was given upon registration. After applying for the study, participants were given access to an online screening survey consisting of demographic questions as well as online versions of the screening and outcome measures. If initial inclusion criteria were met (ie, scoring ≥10 on the QIDS-A17-SR and not meeting any of the exclusion criteria), participants were contacted within the following few days for a diagnostic interview (ie, MINI 7.0) over the phone with study therapists or clinical psychologists in the research group. During this interview, each participant’s identity was confirmed through full name, social security number, and address. If the interview confirmed a current major depressive episode and no exclusion criteria were fulfilled, patients were included. All cases were discussed with the principal investigator and coordinators of the study as well as with a senior psychiatrist to determine inclusion or exclusion. Included participants were randomized to either the treatment or control condition in a 1:1 ratio, the process of which was conducted by an independent researcher using an online randomization tool [29]. See the CONSORT flowchart in Figure 1 for an illustration of the procedure. All participants who were excluded were given this information personally and offered advice or a referral to appropriate care. Neither participants nor therapist, per se, could be blinded to treatment condition. Since only self-report measures were employed after baseline assessment, any further blinding was redundant.

### Interventions

The IPDT intervention consisted of eight therapist-supported self-help modules delivered over 8 weeks on a secure online platform [30]. Modules consisted of texts and videos followed by exercises that participants completed and sent to their therapist upon which they received feedback, typically within 24 hours on working days. In addition, the treatment included one 30-minute chat session between participants and their therapist each week. Chat sessions were unstructured, but therapists were instructed to focus on emotional conflicts underlying depressive symptomatology and relationship events affecting depression, as well as lending support and guidance throughout the treatment. Participants could also contact their therapist at any time via the integrated message system on the platform. Participants that had been inactive for a week received an encouraging message on the platform. Textbox 1 details the content of the eight modules.

**Figure 2:**
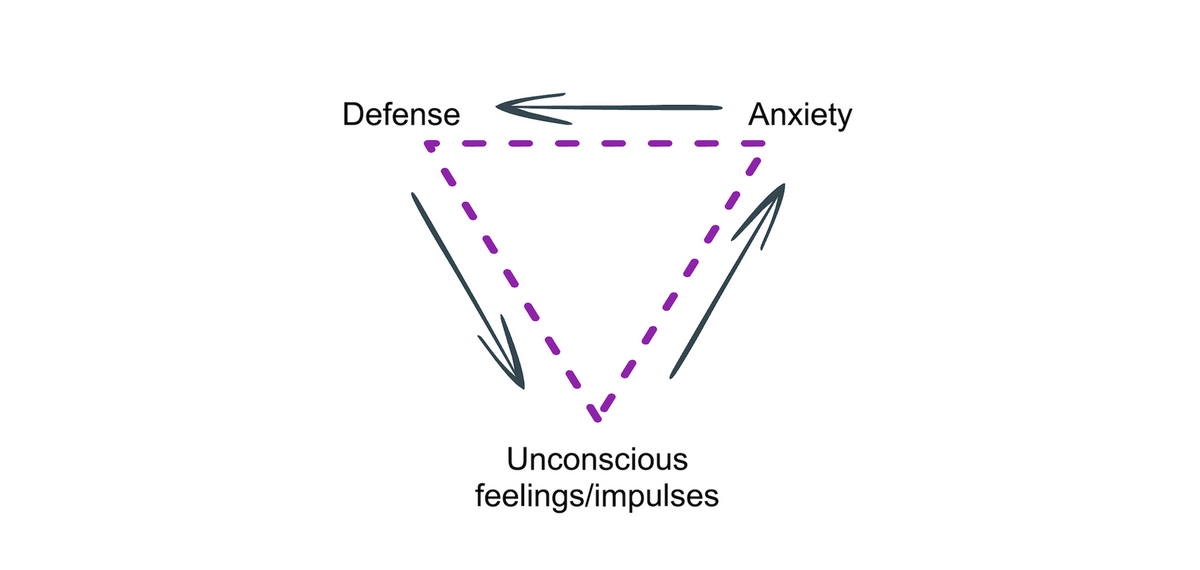
Malan's (1979) triangle of conflict.

The IPDT program was developed specifically for this project (by the first and second author) and was based on similar principles as a treatment program with efficacy for adults in several RCTs (eg, Johansson et al [21] and Zwerenz et al [22]). The aim of the intervention was to decrease emotional avoidance and to increase awareness, experience, and expression of emotions. Following the “triangle of conflict” [31], participants were first introduced to the idea that emotional conflicts may underlie depressive symptoms. According to this theory, feelings that are perceived as threatening to key relationships (ie, when someone important to us reacts to our emotions by expressing discomfort, withdrawal, or by expressing anger) will evoke anxiety and, hence, be suppressed and/or distorted in order to maintain the relationship [32]. The triangle of conflict depicts how these unconscious feelings and/or impulses generate anxiety, which leads to the use of defenses (ie, experiential avoidance) to ward off the anxiety-laden affects and, thus, keep them out of awareness (see Figure 2). However, rigid use of defenses will lead to negative consequences in the long run, such as depression and anxiety as well as hampering our ability to grow and mature. Throughout the treatment participants were encouraged to become aware of their own defenses, notice and regulate anxiety, and gradually approach previously warded-off feelings related to situations that could trigger depressive symptoms. The final part of the program contained material on how to express previously avoided affects in close relationships, with specific attention to psychological developmental issues relevant to adolescence (ie, Blatt [33]). Compared to existing internet-based psychodynamic treatments for adults, the modules were substantially shorter and easier to read, with vignettes that were more recognizable for the age group.

**Treatment modules.**

Module 1: Introducing theory on the interplay between basic emotions and attachment. Emphasis lies on how attachment needs are given priority above our emotions, thus leading to affect phobias (illustrated using the “triangle of conflict”).

Module 2: Superego, shame, and self-compassion. Focus on building the capacity for self-observation and differentiating between old habits of self-neglect and self-criticizing versus more healthy parts of the ego.

Module 3: Differentiation between optimal and too-high levels of anxiety. Anxiety regulation through an increased capacity for self-observation and breathing exercises.

Module 4: Affect theory and the visceral experience of affect. Exposure to warded-off feelings through an expressive writing exercise.

Module 5: Identifying and understanding defensive patterns. Identifying different defensive maneuvers and the long-term negative consequences connected to them.

Module 6: Especially problematic feelings: anger, sadness, and guilt. Mixed and complex emotions. How to notice, accept, and experience them viscerally.

Module 7: Interpersonal patterns of relatedness and self-definition. Identifying one’s predominant relationship patterns according to Sidney Blatt’s theory on anaclitic and introjective polarities of personality [33]. Participants are also taught how this is connected to avoidance of our emotions and how to break these patterns by going against them.

Module 8: Communicating and expressing affects appropriately and identifying and repairing relationship ruptures. Moving forward and maintaining gains.

The control condition consisted of supportive contact over the internet with weekly monitoring of symptoms and well-being. Each week, participants were contacted with a text message by their personally assigned therapist—both licensed clinical psychologists (JM and KL)—typically containing questions regarding their well-being during the previous week. All messages from participants were responded to, typically within 24 hours. Participants could also contact their therapist at any time via the integrated message system on the platform. Therapists provided basic support, expressed empathy and validated emotions and experiences, and encouraged participants to describe and discuss distressing life events but used no psychotherapeutic techniques or interventions. Participants who expressed suicidality, either in weekly ratings or verbally through text, were immediately contacted via phone or text message for an in-depth assessment and were offered appropriate support.

The control condition did not include any treatment material or chat sessions. After 10 weeks, participants in the control condition were offered the IPDT program without chat sessions (not presented in this study).

### Therapists

A total of 11 therapists were included in the project and were either licensed psychologists (n=2) or students of clinical psychology in the last semesters of their psychologist training (n=9). All therapists had chosen to specialize in PDT for their clinical training and, thus, had taken courses in PDT theory and practice. Student therapists received a 1-day training by the treatment developers (JM and KL) and treated the majority of participants in the treatment (n=32). All therapists were supervised in group (n = 5-6) during 90-minute weekly sessions by an experienced psychotherapist (PL) specialized in experiential psychodynamic psychotherapies.

### Instruments

Psychiatric diagnoses at inclusion were assessed during telephone interviews using the MINI 7.0 [28]. The MINI 7.0 interview was modified for adolescents by adding the irritability criteria to the depression module as well as the separation anxiety module from the MINI for Children and Adolescents (MINI KID). The MINI 7.0 is a well-validated diagnostic measure. It has not been validated in a Swedish adolescent population, but a study of an earlier MINI version exists [34]. Furthermore, the suicidality module was replaced by the Columbia-Suicide Severity Rating Scale (C-SSRS) [35], baseline/screening version 090114. The C-SSRS was chosen due to it being more easily administered and also recommended by the United States Food and Drug Administration [36] as the instrument of choice in clinical trials. The C-SSRS has been used in samples of Swedish young adults [37] and validated in a population consisting of Danish adolescents [38].

The diagnostic interviews were conducted by licensed psychologists (n=3) from the research team as well as psychology students (n=3) who received training in both the MINI 7.0 and the C-SSRS.

The primary outcome was self-reported depressive symptoms, measured with the QIDS-A17-SR, a reliable self-rated measure of depressive symptoms validated for both adults and adolescents [27,39]. Using all available data from all time points, an average Cronbach alpha of .76 (range .71-.85) was found, suggesting an acceptable internal consistency. Assessments were made via internet-delivered self-rating forms pretreatment, weekly during treatment, and posttreatment. Furthermore, the treatment group was assessed 6 months following treatment termination.

Secondary outcomes were measured using the Generalized Anxiety Disorder 7-item scale (GAD-7) [40], the Montgomery Åsberg Depression Rating Scale–self-rated (MADRS-S) [41,42], and the Self-Compassion Scale short-form [43], which were administered pre- and posttreatment, as well as the Difficulties in Emotion Regulation Scale [44], which was administered weekly. Cronbach alpha values suggested that internal consistency on secondary outcome measures ranged from acceptable to good (alpha=.76, .82, .71, and .89, respectively). All secondary outcome instruments have been validated in adolescent populations [42,45-47] and official translations were used.

### Statistical Analysis

Sample size was determined a priori based on an expected between-group effect size of *d*=0.70 previously observed for the comparison of ICBT participants and wait-list controls for anxiety and depression in adolescents [11]. At a 5% significance level, 80% power, and *d*=0.70, a total sample size of 72 was needed.

### Primary Outcome

Since the primary outcome was measured weekly within subjects, we applied multilevel modeling (MLM) [48,49] to account for individual changes over time and to test between-group differences in change rates. MLM adequately handles nested data structures and uses all available data from randomized participants, constituting a full intention-to-treat (ITT) analysis. Further, MLM provides unbiased estimates with a relatively unrestrictive assumption about missing data (ie, missing at random [50]). Model building started with estimating a basic time model that included random intercepts and fixed and random slopes for *time*. *Time* was coded as 0 for pretreatment, 1-9 for the weekly assessments during treatment, and 10 for the follow-up assessment point. To account for possible nonlinearity in the data, a quadratic term for *time* (ie, *time* × *time*) was also tested. It was found significant as a main effect as well as improving model fit for both measures (ie, reduction in Akaike’s Information Criterion >2) and was, thus, retained; a random quadratic effect was discarded since the model did not converge. Lastly, *group*—coded as 0 for control and 1 for IPDT—was entered both as a main effect, to test for possible differences between IPDT and control groups at pretreatment assessment, as well as in interaction with *time*, to test for group differences in change rates over time. All models were fitted with maximum-likelihood estimation and an unstructured covariance structure for the random effects. The MLM analyses were done using SPSS, version 25.0.0.1. (IBM Corp).

### Secondary Outcomes

For all secondary outcome measures, missing data at posttreatment (n=3) were addressed using multiple imputation in R, version 1.7 (The R Foundation), packages Mice [51] and Miceadds [52]. Between-group differences in the secondary measures were determined by an analysis of covariance (ANCOVA), controlling for individual differences on the respective measures at baseline.

Between-group effect size for the primary outcome was calculated using model-estimated means at posttreatment and the observed pretreatment sample SD, as recommended by Feingold [53]. For secondary measures, between-group effect sizes were transformed from eta2 from the ANCOVA, according to the formula described by Cohen [54].

### Response and Remission Rates

Response to treatment was calculated using the Reliable Change Index (RCI) [55] and was defined as fulfilling the RCI criteria while scoring 2 SD below the pretreatment mean. Baseline Cronbach alpha from this study was used. Remission was defined as a QIDS-A17-SR score of 6 or below [39].

## Results

### Participants

A total of 76 participants were included in the study (IPDT 38/76, 50%; control 38/76, 50%). Out of 76 participants, 61 (80%) were female and the sample had a mean age of 16.6 years (SD 1.1). A total of 4 participants randomized to IPDT never entered treatment, meaning that they never participated in any of the exercises or chat sessions and had no contact with the therapist, except in 2 cases to say that they wanted to drop out. A total of 3 of these participants never opened the initial study message, meaning that they dropped out without knowing their allocation (ie, treatment or control). These 4 nonstarters were still included in the ITT analysis for the primary outcome measure, but not for the secondary outcome measures.

Patients’ characteristics for the full ITT sample are presented in Table 1. All reported psychotropic medications were selective serotonin reuptake inhibitors (SSRIs) with dosages stable for at least 3 months. A total of 2 of the 4 patients on SSRIs at baseline also had prescriptions for anxiety medication—1 prometazin and 1 hydrocizine—and a sleeping aid (ie, melatonin) when needed. A total of 30 participants out of 76 (39%) reported living in a large city, 28 (37%) reported living in a smaller town, and 18 (24%) reported living in a rural area. A total of 73 participants out of 76 (96%) reported going to school. Out of 76 participants, 47 (62%) reported living with both parents; 23 (30%) reported living with either one parent, moving between two parents, or living with a remarried parent; and 6 (8%) reported living alone or with a friend, sibling, or partner.

**Table 1:** Demographic data at baseline^a^.

Characteristic		IPDT^b^ group (n=38)	Control group (n=38)
**Gender, n (%)**			
	Female	31 (82)	30 (79)
	Uncertain or other	4 (11)	0 (0)
Age (years), mean (SD)		16.6 (1.11)	16.5 (1.13)
**Disorder, n (%)**			
	Major depressive disorder^c^	38 (100)	38 (100)
	Any anxiety disorder^c^	22 (58)	23 (62)
	Posttraumatic stress disorder^c^	4 (11)	1 (3)
	Eating disorder^c,d^	2 (5)	1 (3)
**Self-harm behavior, n (%)**			
	Ever	20 (53)	10 (26)
	Current	9 (24)	4 (11)
Currently on psychotropic medication, n (%)		2 (5)	2 (5)

^a^There were no significant between-group differences on any of the data at baseline.

^b^IPDT: internet-based psychodynamic therapy.

^c^Confirmed by the Mini International Neuropsychiatric Interview 7.0 (MINI 7.0).

^d^Bulimia nervosa and binge-eating disorder.

### Primary Outcome

**Figure 3:**
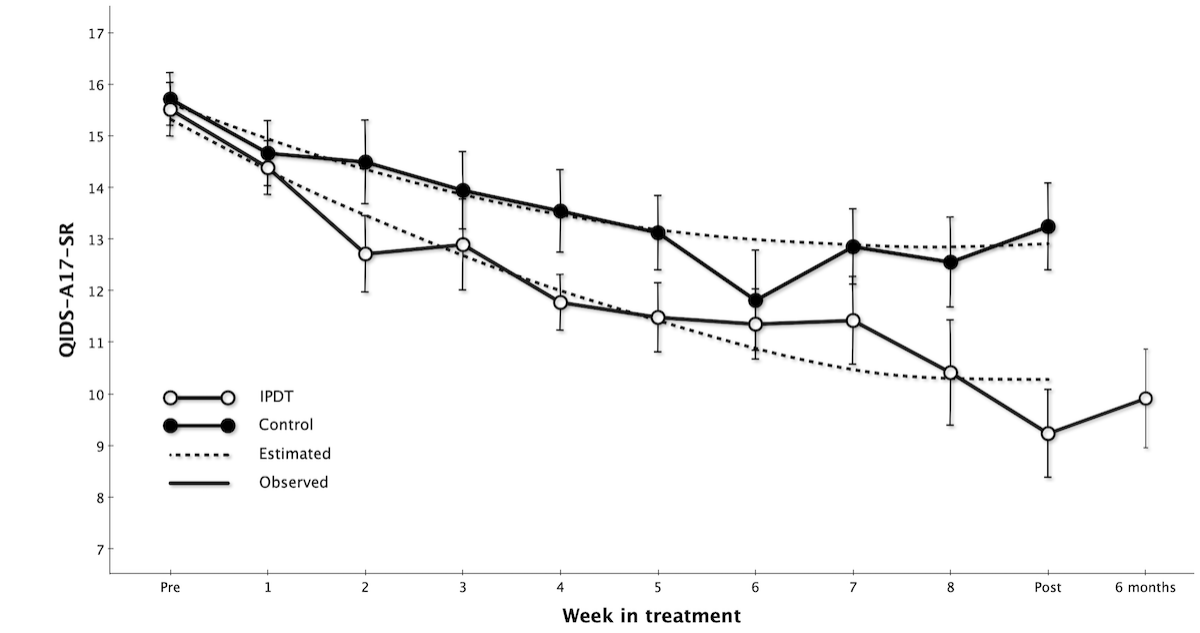
Weekly mean scores on the QIDS-A17-SR (Quick Inventory of Depressive Symptomatology for Adolescents). IPDT: internet-based psychodynamic therapy.

Fixed-effect estimates from our MLM analysis for QIDS-A17-SR are displayed in Table 2. The model intercept (15.62) represents the average baseline score in the control group while the IPDT estimate (–0.32) represents the group difference at baseline between IPDT and control groups, which was nonsignificant (*P*=.67). The slope estimate (–0.74) represents the initial symptom reduction rate in the control group and the *time* × *time* (estimate=0.05) means that for each session, symptoms were reduced at a slightly slower rate. The IPDT versus control estimate (–0.29) represents the additional weekly decrease in symptoms for patients in the IPDT group. This term proved significant (*P*=.01), indicating that patients in the IPDT group had a significantly steeper decline in symptoms compared to those in the control group. The between-group effect size at the posttreatment assessment point was large (*d*=0.82, 95% CI 0.35-1.29) and in favor of IPDT. The observed and estimated change on the QIDS-A17-SR is illustrated in Figure 3.

### Secondary Outcomes

The ANCOVAs revealed that improvements on all secondary measures were significantly greater in the treatment group. Detailed results and effect sizes are presented in Table 3.

**Table 2:** Multilevel models estimating changes over time in the primary outcome measure, QIDS-A17-SR^a^.

Model estimates		Estimate	95% CI	*P* value
**Baseline score**				
	Intercept	15.62	14.57 to 16.68	<.001
	IPDT^b^	–0.32	–1.76 to 1.13	.67
**Rate of change**				
	Slope	–0.73	–1.01 to –0.46	<.001
	Time × time	0.05	0.02 to 0.07	<.001
	IPDT versus control	–0.29	–0.51 to –0.07	.01
**Variance components**				
	Residual variance	6.10	5.40 to 6.89	<.001
	Intercept	7.61	5.04 to 11.50	<.001
	Slope	0.13	0.08 to 0.23	<.001
	Correlation	–0.05	–0.29 to 0.38	.78
Between-group effect size (Cohen *d*)		0.82	0.35 to 1.29	

^a^QIDS-A17-SR: Quick Inventory of Depressive Symptomatology for Adolescents.

^b^IPDT: internet-based psychodynamic therapy.

**Table 3:** Secondary outcome results.

Measure		Pretreatment, mean (SD)	Posttreatment, mean (SD)	Follow-up, mean (SD)	Posttreatment, between-group Cohen *d* (95% CI)	*P* value
**MADRS-S^a^**						
	IPDT^b^	24.97 (7.06)	18.97 (7.53)	N/A^c^	0.80 (0.32-1.28)	<.001
	Control^d^	26.21 (6.93)	25.84 (8.51)			
**GAD-7^e^**						
	IPDT	12.35 (4.11)	8.18 (4.62)	8.41 (5.54)	0.78 (0.30-1.26)	<.001
	Control	10.45 (3.88)	10.42 (4.65)			
**DERS^f^**						
	IPDT	55.44 (12.12)	41.53 (14.47)	N/A	0.97 (0.48-1.46)	<.001
	Control	56.42 (10.64)	53.0 (14.41)			
**SCS-SF^g^**						
	IPDT	25.91 (5.68)	31.06 (7.31)	N/A	0.65 (0.18-1.12)	.003
	Control	26.87 (5.65)	27.08 (7.69)			

^a^MADRS-S: Montgomery Åsberg Depression Rating Scale–self-rated.

^b^IPDT: internet-based psychodynamic therapy, n=34.

^c^Not applicable.

^d^Control, n=38.

^e^GAD-7: Generalized Anxiety Disorder 7-item scale.

^f^DERS: Difficulties in Emotion Regulation Scale.

^g^SCS-SF: Self-Compassion Scale short-form.

### Response and Remission Rates

In all analyses of response and remission rates, missing cases (n=2, 1 in each group) were categorized as not improved. Using fulfillment of RCI and scoring 2 SDs below the pretreatment mean as criteria for response, 56% (19/34) of the treatment group compared to 21% (8/38) of the control group were categorized as responders. There was a significant difference between the groups in favor of the treatment group (χ^2^_4_=10.9, *P*=.03). Using a QIDS-A17-SR score of 6 or under as a cutoff for remission, 35% (12/34) of the treatment group compared to 8% (3/38) of the control group were categorized as remitted. A higher proportion of participants in the IPDT group fulfilled the criteria for remission (χ^2^_1_=8.2, *P*=.004).

### Follow-Up Assessment

Follow-up assessments at 6 months posttreatment were conducted using the QIDS-A17-SR and GAD-7. Data were obtained from all participants who entered the treatment (n=34). Pairwise *t* tests between post- and follow-up assessments indicated no significant differences for either measure (QIDS-A17-SR mean difference −0.61, t_32_=−0.66, *P*=.51; GAD-7 mean difference -0.09, t_32_=−0.12, *P*=.91), suggesting results for depression and anxiety were maintained during the follow-up period.

### Negative Effects and Adverse Events

Posttreatment QIDS-A17-SR scores and an open-ended question concerning negative effects [56] were used to assess potential negative effects during the trial. No participant in the treatment condition deteriorated reliably on the QIDS-A17-SR, while this was true for 3 participants in the control condition.

When responding to the open-ended question regarding negative effects, the vast majority did not report any (28/34, 82%). Out of 34 participants, 1 (3%) participant described that online text-based interactions were not optimal due to feelings of loneliness, and another participant (3%) described increased awareness of feelings of anger and that this was painful and distressing in the short term; however, the same participant then described the mastery of these angry feelings gained through the treatment as positive in the long term. Out of 34 participants, 2 (6%) described feelings of distress in connection with facing previously avoided thoughts and feelings, and 2 (6%) participants found the treatment format stressful; 1 (3%) of these participants also described feelings of shame in connection with not completing exercises on time. Thus, 6 patients in total out of 34 (18%) reported negative effects of the treatment. No serious adverse events were reported during the trial.

### Program Use and Treatment Acceptability

Of the 34 participants that entered treatment, 4 (12%) dropped out of treatment, meaning that they stopped opening modules, attending chat sessions, or responding to messages before week 7. The completion of modules was defined as completing at least one exercise in the module. The mean number of completed modules was 5.8 (SD 2.4) of the 8 that were available. Excluding the 4 dropouts, the mean number was 6.2 (SD 1.9) and the median was 7. The mean number of chat sessions attended was 6.6 (SD 2.1) of the 8 available. Excluding the 4 dropouts, the mean number of chat sessions attended was 7.1 (SD 1.4). Of the 34 participants, 1 (3%) who did not drop out completed zero modules but continued attending chat sessions throughout the treatment period. Spearman correlations showed no dose-response relationship.

## Discussion

### Principal Findings

This RCT aimed to evaluate an affect-focused psychodynamic internet-based treatment for depression in a sample of adolescents (15-18 years of age). The results indicated that the IPDT treatment was effective in reducing depression and anxiety, as well as in enhancing emotion regulation and self-compassion, compared to a supportive contact control condition. Results indicated that the treatment facilitated clinically significant changes [55] as 56% of the treatment group recovered according to RCI and 35% scored below clinical cutoff on the primary outcome measure after completing the treatment. Treatment gains for depression and anxiety symptoms were maintained at the 6-month follow-up. In addition, the IPDT treatment seemed acceptable and tolerable overall, although some distress was reported related to taking part in the treatment. None of the participants deteriorated reliably during the intervention. The vast majority of patients (28/34, 82%) who entered treatment did not report any negative effects, and those that were reported were relatively mild. The occurrence and nature of negative experiences were similar to what has previously been reported for ICBT in treatment with adults and adolescents [14,56]. The absence of deterioration and negative effects is especially important considering that deterioration seems common among young people in psychotherapy [57].

Between-group effect sizes were similar to those found in previous trials on IPDT targeting adult psychopathology [21] as well as ICBT targeting adolescent depression [11,14,15]. The significant result for anxiety has not been found in ICBT treatments in previous trials for the same target group [14,15] and this is a promising finding, albeit in need of replication. Although the target groups appear similar, there might be population differences affecting these results. Previous trials have used another anxiety measure, rendering comparisons of initial anxiety levels difficult. However, the results are in line with those shown in a meta-analysis by Driessen et al [16] who found that short-term PDT targeting depression was significantly more efficacious than other treatments on anxiety measures. One reason for this might be the more transdiagnostic nature of PDT, which, although focused on depression symptoms to a certain extent, might target a wider scope of symptoms. The model for depression in affect-focused psychotherapy is based on the triangle of conflict, which is the same across different psychiatric disorders [31].

Although some of the more classical ingredients of psychodynamic therapy are lost in the translation to a guided self-help program, being significantly more directive and not including transference work to the same extent, the treatment is clearly based on a psychodynamic model. This is an important point since this distinguishes the treatment from ones that have been previously developed and assessed for adolescent depression. The model for understanding depression, which was also presented to the participants, is the triangle of conflict [31]. Participants are taught about attachment theory and are encouraged to reflect on their own patterns of relating based on this and whether there are any patterns they would like to change. Furthermore, participants are encouraged to reflect on and experience affects that have been perceived as threatening to key attachment relationships. The treatment relies heavily on working with defenses against these anxiety-laden affects, leading to exposure to painful emotions [32]. Furthermore, the therapists were instructed not to give advice, but rather to invite exploration and to interpret problems using the triangle of conflict. In ICBT for adolescents, chat sessions are more structured, while the chat sessions in this RCT’s treatment were unstructured and the participants chose the subject; however, therapists were instructed to be symptom-focused, as the primary aim of the treatment was to uncover inner emotional conflicts creating and perpetuating symptoms of depression. While including exercises in psychodynamic treatments is not common practice, this is included in some psychodynamic modalities when deemed appropriate [58]. A future study will aim to examine the nature of therapist interventions in chat sessions during the treatment in detail. Future studies should also address whether these different treatments may be suitable for different target groups of depressed adolescents.

This study did not comprise an evaluation of adding synchronous chat sessions to the standard guided self-help format, so it is not possible to separate out the impact of each element to the overall effectiveness of the intervention. However, some aspect of the positive results may be related to the text-chat component, as it might have increased motivation and hence continued participation in the treatment. In order to minimize the workload, therapists were permitted to have two parallel chat sessions at the same time. The added value of synchronous chat sessions is an area for further research in the future.

While the chat sessions make the treatment format more time-consuming compared to internet-based interventions targeting adult depression, it is comparable to some other internet-based treatment protocols targeting adolescents [14,15,59] that enhance treatment protocols with blended real-time elements (ie, chat for 30-45 minutes or 20 minutes of weekly phone calls for 14 weeks, respectively). Also, 8 weeks of treatment is substantially shorter than other existing psychodynamic face-to-face protocols in the treatment of adolescent depression [20]. The rates of participation and completion of modules were lower than studies on adults typically show [60]. However, studies of face-to-face treatments for adolescents generally report high levels of attrition (eg, Abbass et al [19]).

The sample of the study is predominantly female. This is in line with other psychotherapy trials on face-to-face treatment (eg, Abbass et al [19]) as well as internet interventions [61]. There is also some evidence that the female predominance is even larger for internet-based interventions than in those conducted face to face [61]. One suggested reason for that is that males tend to need more influence from family and/or peers in order to seek support, and that seeking internet-based interventions relies more on individual motivation and choice. The dominance of females in the study sample could be seen as a problem, although common in the field of psychotherapy research, with generalizability of the results. It is clear that an important challenge for future treatments as well as trials is to find ways to reach and motivate males to apply as well.

### Strengths and Limitations

Apart from the randomized design, a considerable strength is that a “pure” wait list was not used [62]. Instead, IPDT was compared to weekly support. In addition, a structured diagnostic interview was carried out before inclusion to confirm the depression diagnosis. The study approach was to use weekly measures of the primary outcome to detect group differences, which allowed for more sophisticated analyses. Furthermore, the trial had a low rate of attrition both at the postassessment and follow-up measurements, increasing the reliability of the results. The sample in this trial is representative of the depressed adolescent population and is a clearly psychiatric one. Participants had relatively high comorbidity and a substantial amount had current and/or past self-harm behaviors. The exclusion of severely suicidal teenagers is also in line with other research in the field (eg, Sander et al [63]).

Limitations of the study include the fact that no diagnostic interviews were conducted after the treatment and that 6-month follow-up data were only collected for two of the outcome measures. The reason for this was to avoid burdening the young people with too many measures and interviews, as it might have heightened the risk of missing data. Therefore, the most relevant instruments were prioritized for both postassessment and follow-up measurements. Also, although all interviewers were trained in the application of the MINI 7.0 and the C-SSRS, no measures of interrater reliability were conducted as interviews were not recorded. All measures in the study have been validated in adolescent populations and official translations were used. However, except for the MADRS-S [42], they have not been validated in Swedish adolescent populations. The lack of validation studies for teenage populations is a common problem in the field and one future aim is to publish psychometric data for the Swedish translations of the questionnaires. As shown, they have acceptable-to-good internal consistency in this study’s sample.

### Conclusions

This RCT’s results provide preliminary support for affect-focused IPDT for adolescent depression. This study furthers the evidence that psychodynamic models can be translated into internet-based treatments and strengthens the evidence for the effectiveness of psychodynamic treatments in general.

Future research could aim to explore whether this treatment, in an adapted format, would be suitable for a transdiagnostic sample or to target adolescent anxiety. This study is preceding a noninferiority trial comparing IPDT to the already-proven effective ICBT program for depression in adolescents [14,15]. Furthermore, future research should address the efficacy of the treatment in other settings (ie, other countries).

## Appendix

Multimedia Appendix 1 CONSORT eHEALTH checklist (V 1.6.1).

## Abbreviations

ANCOVA: analysis of covariance
CBT: cognitive behavioral therapy
CONSORT: Consolidated Standards of Reporting Trials
C-SSRS: Columbia-Suicide Severity Rating Scale
DSM-5: Diagnostic and Statistical Manual of Mental Disorders, Fifth Edition
GAD-7: Generalized Anxiety Disorder 7-item scale
ICBT: internet-based cognitive behavioral therapy
IPDT: internet-based psychodynamic therapy
ISRCTN: International Standard Randomised Controlled Trial Number
ITT: intention-to-treat
MADRS-S: Montgomery Åsberg Depression Rating Scale–self-rated
MINI 7.0: Mini International Neuropsychiatric Interview 7.0
MINI KID: Mini International Neuropsychiatric Interview for Children and Adolescents
MLM: multilevel modelling
PDT: psychodynamic psychotherapy
QIDS-A17-SR: Quick Inventory of Depressive Symptomatology for Adolescents
RCI: Reliable Change Index
RCT: randomized controlled trial
SSRI: selective serotonin reuptake inhibitor

## References

[1] (2019). World Health Organization.

[2] Alaie I, Philipson A, Ssegonja R, Hagberg L, Feldman I, Sampaio F, Möller M, Arinell H, Ramklint M, Päären A, von Knorring L, Olsson G, von Knorring A, Bohman H, Jonsson U (2019). Uppsala Longitudinal Adolescent Depression Study (ULADS). BMJ Open.

[3] Essau CA (2005). Frequency and patterns of mental health services utilization among adolescents with anxiety and depressive disorders. Depress Anxiety.

[4] Rocha TB, Graeff-Martins AS, Kieling C, Rohde LA (2015). Provision of mental healthcare for children and adolescents: A worldwide view. Curr Opin Psychiatry.

[5] Gulliver A, Griffiths KM, Christensen H (2010). Perceived barriers and facilitators to mental health help-seeking in young people: A systematic review. BMC Psychiatry.

[6] Andersson G, Titov N, Dear BF, Rozental A, Carlbring P (2019). Internet-delivered psychological treatments: From innovation to implementation. World Psychiatry.

[7] Foroushani PS, Schneider J, Assareh N (2011). Meta-review of the effectiveness of computerised CBT in treating depression. BMC Psychiatry.

[8] Salzer S, Stefini A, Kronmüller KT, Leibing E, Leichsenring F, Henningsen P, Peseschkian H, Reich G, Rosner R, Ruhl U, Schopf Y, Steinert C, Vonderlin E, Steil R (2018). Cognitive-behavioral and psychodynamic therapy in adolescents with social anxiety disorder: A multicenter randomized controlled trial. Psychother Psychosom.

[9] Andersson G, Carlbring P, Titov N, Lindefors N (2019). Internet interventions for adults with anxiety and mood disorders: A narrative umbrella review of recent meta-analyses. Can J Psychiatry.

[10] Vigerland S, Lenhard F, Bonnert M, Lalouni M, Hedman E, Ahlen J, Olén O, Serlachius E, Ljótsson B (2016). Internet-delivered cognitive behavior therapy for children and adolescents: A systematic review and meta-analysis. Clin Psychol Rev.

[11] Ebert DD, Zarski A, Christensen H, Stikkelbroek Y, Cuijpers P, Berking M, Riper H (2015). Internet and computer-based cognitive behavioral therapy for anxiety and depression in youth: A meta-analysis of randomized controlled outcome trials. PLoS One.

[12] Grist R, Croker A, Denne M, Stallard P (2019). Technology delivered interventions for depression and anxiety in children and adolescents: A systematic review and meta-analysis. Clin Child Fam Psychol Rev.

[13] Ye X, Bapuji SB, Winters SE, Struthers A, Raynard M, Metge C, Kreindler SA, Charette CJ, Lemaire JA, Synyshyn M, Sutherland K (2014). Effectiveness of internet-based interventions for children, youth, and young adults with anxiety and/or depression: A systematic review and meta-analysis. BMC Health Serv Res.

[14] Topooco N, Berg M, Johansson S, Liljethörn L, Radvogin E, Vlaescu G, Nordgren LB, Zetterqvist M, Andersson G (2018). Chat- and internet-based cognitive-behavioural therapy in treatment of adolescent depression: Randomised controlled trial. BJPsych Open.

[15] Topooco N, Byléhn S, Dahlström Nysäter E, Holmlund J, Lindegaard J, Johansson S, Åberg L, Bergman Nordgren L, Zetterqvist M, Andersson G (2019). Evaluating the efficacy of internet-delivered cognitive behavioral therapy blended with synchronous chat sessions to treat adolescent depression: Randomized controlled trial. J Med Internet Res.

[16] Driessen E, Hegelmaier LM, Abbass AA, Barber JP, Dekker JJ, Van HL, Jansma EP, Cuijpers P (2015). The efficacy of short-term psychodynamic psychotherapy for depression: A meta-analysis update. Clin Psychol Rev.

[17] Keefe JR, McCarthy KS, Dinger U, Zilcha-Mano S, Barber JP (2014). A meta-analytic review of psychodynamic therapies for anxiety disorders. Clin Psychol Rev.

[18] Midgley N, O’Keeffe S, French L, Kennedy E (2017). Psychodynamic psychotherapy for children and adolescents: An updated narrative review of the evidence base. J Child Psychother.

[19] Abbass AA, Rabung S, Leichsenring F, Refseth JS, Midgley N (2013). Psychodynamic psychotherapy for children and adolescents: A meta-analysis of short-term psychodynamic models. J Am Acad Child Adolesc Psychiatry.

[20] Goodyer IM, Reynolds S, Barrett B, Byford S, Dubicka B, Hill J, Holland F, Kelvin R, Midgley N, Roberts C, Senior R, Target M, Widmer B, Wilkinson P, Fonagy P (2017). Cognitive behavioural therapy and short-term psychoanalytical psychotherapy versus a brief psychosocial intervention in adolescents with unipolar major depressive disorder (IMPACT): A multicentre, pragmatic, observer-blind, randomised controlled superiority trial. Lancet Psychiatry.

[21] Johansson R, Björklund M, Hornborg C, Karlsson S, Hesser H, Ljótsson B, Rousseau A, Frederick RJ, Andersson G (2013). Affect-focused psychodynamic psychotherapy for depression and anxiety through the internet: A randomized controlled trial. PeerJ.

[22] Zwerenz R, Becker J, Johansson R, Frederick RJ, Andersson G, Beutel ME (2017). Transdiagnostic, psychodynamic Web-based self-help intervention following inpatient psychotherapy: Results of a feasibility study and randomized controlled trial. JMIR Ment Health.

[23] Johansson R, Hesslow T, Ljótsson B, Jansson A, Jonsson L, Färdig S, Karlsson J, Hesser H, Frederick RJ, Lilliengren P, Carlbring P, Andersson G (2017). Internet-based affect-focused psychodynamic therapy for social anxiety disorder: A randomized controlled trial with 2-year follow-up. Psychotherapy (Chic).

[24] Johansson R, Ekbladh S, Hebert A, Lindström M, Möller S, Petitt E, Poysti S, Larsson MH, Rousseau A, Carlbring P, Cuijpers P, Andersson G (2012). Psychodynamic guided self-help for adult depression through the internet: A randomised controlled trial. PLoS One.

[25] Andersson G, Paxling B, Roch-Norlund P, Östman G, Norgren A, Almlöv J, Georén L, Breitholtz E, Dahlin M, Cuijpers P, Carlbring P, Silverberg F (2012). Internet-based psychodynamic versus cognitive behavioral guided self-help for generalized anxiety disorder: A randomized controlled trial. Psychother Psychosom.

[26] Schulz K, Altman D, Moher D, CONSORT Group (2010). CONSORT 2010 statement: Updated guidelines for reporting parallel group randomised trials. BMJ.

[27] Bernstein IH, Rush AJ, Trivedi MH, Hughes CW, Macleod L, Witte BP, Jain S, Mayes TL, Emslie GJ (2010). Psychometric properties of the Quick Inventory of Depressive Symptomatology in adolescents. Int J Methods Psychiatr Res.

[28] Sheehan DV, Lecrubier Y, Sheehan KH, Amorim P, Janavs J, Weiller E, Hergueta T, Baker R, Dunbar GC (1998). The Mini-International Neuropsychiatric Interview (M.I.N.I.): The development and validation of a structured diagnostic psychiatric interview for DSM-IV and ICD-10. J Clin Psychiatry.

[29] RANDOM.ORG.

[30] Vlaescu G, Alasjö A, Miloff A, Carlbring P, Andersson G (2016). Features and functionality of the Iterapi platform for internet-based psychological treatment. Internet Interv.

[31] Malan DH (1979). Individual Psychotherapy and the Science of Psychodynamics.

[32] Fosha D (2001). The dyadic regulation of affect. J Clin Psychol.

[33] Blatt SJ (2008). Polarities of Experience: Relatedness and Self-Definition in Personality Development, Psychopathology, and the Therapeutic Process.

[34] Högberg C, Billstedt E, Björck C, Björck PO, Ehlers S, Gustle L, Hellner C, Höök H, Serlachius E, Svensson MA, Larsson J (2019). Diagnostic validity of the MINI-KID disorder classifications in specialized child and adolescent psychiatric outpatient clinics in Sweden. BMC Psychiatry.

[35] Posner K, Brown GK, Stanley B, Brent DA, Yershova KV, Oquendo MA, Currier GW, Melvin GA, Greenhill L, Shen S, Mann JJ (2011). The Columbia-Suicide Severity Rating Scale: Initial validity and internal consistency findings from three multisite studies with adolescents and adults. Am J Psychiatry.

[36] (2012). Guidance for Industry. Suicidal Ideation and Behavior: Prospective Assessment of Occurrence in Clinical Trials.

[37] Beckman K, Lindh AU, Waern M, Stromsten L, Renberg ES, Runeson B, Dahlin M (2019). Impulsive suicide attempts among young people: A prospective multicentre cohort study in Sweden. J Affect Disord.

[38] Conway PM, Erlangsen A, Teasdale TW, Jakobsen IS, Larsen KJ (2017). Predictive validity of the Columbia-Suicide Severity Rating Scale for short-term suicidal behavior: A Danish study of adolescents at a high risk of suicide. Arch Suicide Res.

[39] Rush AJ, Trivedi MH, Ibrahim HM, Carmody TJ, Arnow B, Klein DN, Markowitz JC, Ninan PT, Kornstein S, Manber R, Thase ME, Kocsis JH, Keller MB (2003). The 16-Item Quick Inventory of Depressive Symptomatology (QIDS), clinician rating (QIDS-C), and self-report (QIDS-SR): A psychometric evaluation in patients with chronic major depression. Biol Psychiatry.

[40] Kroenke K, Spitzer RL, Williams JB, Löwe B (2010). The Patient Health Questionnaire Somatic, Anxiety, and Depressive Symptom Scales: A systematic review. Gen Hosp Psychiatry.

[41] Svanborg P, Asberg M (1994). A new self-rating scale for depression and anxiety states based on the Comprehensive Psychopathological Rating Scale. Acta Psychiatr Scand.

[42] Ntini I, Vadlin S, Olofsdotter S, Ramklint M, Nilsson KW, Engström I, Sonnby K (2020). The Montgomery and Åsberg Depression Rating Scale: Self-assessment for use in adolescents: An evaluation of psychometric and diagnostic accuracy. Nord J Psychiatry.

[43] Raes F, Pommier E, Neff KD, Van Gucht D (2011). Construction and factorial validation of a short form of the Self-Compassion Scale. Clin Psychol Psychother.

[44] Bjureberg J, Ljótsson B, Tull MT, Hedman E, Sahlin H, Lundh L, Bjärehed J, DiLillo D, Messman-Moore T, Gumpert CH, Gratz KL (2016). Development and validation of a brief version of the Difficulties in Emotion Regulation Scale: The DERS-16. J Psychopathol Behav Assess.

[45] Tiirikainen K, Haravuori H, Ranta K, Kaltiala-Heino R, Marttunen M (2019). Psychometric properties of the 7-item Generalized Anxiety Disorder Scale (GAD-7) in a large representative sample of Finnish adolescents. Psychiatry Res.

[46] Cunha M, Xavier A, Castilho P (2016). Understanding self-compassion in adolescents: Validation study of the Self-Compassion Scale. Pers Individ Dif.

[47] Neumann A, van Lier PA, Gratz KL, Koot HM (2010). Multidimensional assessment of emotion regulation difficulties in adolescents using the Difficulties in Emotion Regulation Scale. Assessment.

[48] Singer JD, Willett JB (2003). Applied Longitudinal Data Analysis: Modeling Change and Event Occurrence.

[49] Gueorguieva R, Krystal JH (2004). Move over ANOVA: Progress in analyzing repeated-measures data and its reflection in papers published in the Archives of General Psychiatry. Arch Gen Psychiatry.

[50] Enders CK (2011). Analyzing longitudinal data with missing values. Rehabil Psychol.

[51] van Buuren S, Groothuis-Oudshoorn K (2011). mice: Multivariate imputation by chained equations in R. J Stat Softw.

[52] Robitzsch A, Grund S (2020). miceadds: Some Additional Multiple Imputation Functions, Especially for 'mice'.

[53] Feingold A (2009). Effect sizes for growth-modeling analysis for controlled clinical trials in the same metric as for classical analysis. Psychol Methods.

[54] Cohen J (1988). Statistical Power Analysis for the Behavioral Sciences. 2nd edition.

[55] Jacobson NS, Truax P (1991). Clinical significance: A statistical approach to defining meaningful change in psychotherapy research. J Consult Clin Psychol.

[56] Rozental A, Castonguay L, Dimidjian S, Lambert M, Shafran R, Andersson G, Carlbring P (2018). Negative effects in psychotherapy: Commentary and recommendations for future research and clinical practice. BJPsych Open.

[57] Warren JS, Nelson PL, Mondragon SA, Baldwin SA, Burlingame GM (2010). Youth psychotherapy change trajectories and outcomes in usual care: Community mental health versus managed care settings. J Consult Clin Psychol.

[58] Levenson H (2017). Brief Dynamic Therapy. 2nd edition.

[59] Stjerneklar S, Hougaard E, McLellan LF, Thastum M (2019). A randomized controlled trial examining the efficacy of an internet-based cognitive behavioral therapy program for adolescents with anxiety disorders. PLoS One.

[60] Karyotaki E, Kleiboer A, Smit F, Turner DT, Pastor AM, Andersson G, Berger T, Botella C, Breton JM, Carlbring P, Christensen H, de Graaf E, Griffiths K, Donker T, Farrer L, Huibers MJ, Lenndin J, Mackinnon A, Meyer B, Moritz S, Riper H, Spek V, Vernmark K, Cuijpers P (2015). Predictors of treatment dropout in self-guided Web-based interventions for depression: An 'individual patient data' meta-analysis. Psychol Med.

[61] Rickwood D, Webb M, Kennedy V, Telford N (2016). Who are the young people choosing Web-based mental health support? Findings from the implementation of Australia's national Web-based youth mental health service, eheadspace. JMIR Ment Health.

[62] Cuijpers P, Cristea IA (2016). How to prove that your therapy is effective, even when it is not: A guideline. Epidemiol Psychiatr Sci.

[63] Sander L, Gerhardinger K, Bailey E, Robinson J, Lin J, Cuijpers P, Mühlmann C (2020). Suicide risk management in research on internet-based interventions for depression: A synthesis of the current state and recommendations for future research. J Affect Disord.

